# Who Should Play a Key Role in Preventing Common Mental Disorders that Affect Employees in the Workplace? Results of a Survey with Occupational Health Physicians, Primary Care Physicians, Psychotherapists, and Human Resource Managers

**DOI:** 10.3390/ijerph16081383

**Published:** 2019-04-17

**Authors:** Martina Michaelis, Elisabeth Maria Balint, Florian Junne, Stephan Zipfel, Harald Gündel, Rahna Lange, Monika A. Rieger, Eva Rothermund

**Affiliations:** 1 Institute of Occupational and Social Medicine and Health Services Research, University Hospital Tübingen, 72076 Tübingen, Germany; rahna.lange@gmail.com (R.L.); monika.rieger@med.uni-tuebingen.de (M.A.R.); 2 Research Centre for Occupational and Social Medicine (FFAS), 79098 Freiburg, Germany; 3 Ulm University Medical Center, Department of Psychosomatic Medicine and Psychotherapy, 89070 Ulm, Germany; elisabeth.balint@uniklinik-ulm.de (E.M.B.); harald.guendel@uniklinik-ulm.de (H.G.); eva.rothermund@uniklinik-ulm.de (E.R.); 4 Leadership Personality Center Ulm (LPCU), Ulm University, 89073 Ulm, Germany; 5 Department of Psychosomatic Medicine and Psychotherapy, University Hospital Tübingen, 72076 Tübingen, Germany; florian.junne@med.uni-tuebingen.de (F.J.); stephan.zipfel@med.uni-tuebingen.de (S.Z.)

**Keywords:** common mental disorders, employees, commitment to prevention, survey, occupational health physicians, primary care physicians, psychotherapists, human resource managers

## Abstract

The rising burden of common mental disorders (CMDs) in employees requires strategies for prevention. No systematic data exist about how those involved perceive their roles, responsibilities, and interactions with other professional groups. Therefore, we performed a multi-professional standardized survey with health professionals in Germany. A self-administered questionnaire was completed by 133 occupational health physicians (OHPs), 136 primary care physicians (PCPs), 186 psychotherapists (PTs), and 172 human resource managers (HRMs). Inter alia, they were asked which health professionals working in the company health service and in the outpatient care or in the sector of statutory insurance agents should play a key role in the primary, secondary, and tertiary prevention of CMDs in employees. The McNemar test was used in order to compare the attributed roles among the professionals involved. With regard to CMDs, all the professional groups involved in this study declared OHPs as the most relevant pillar in the field of prevention. In primary prevention, HRMs regarded themselves, OHPs, and health insurance agents as equally relevant in terms of prevention. PTs indicated an important role for employee representatives in this field. In secondary prevention, PCPs were regarded as important as OHPs. HRMs indicated themselves as equally important as OHPs and PCPs. In tertiary prevention, only OHPs identified themselves as main protagonists. The other groups marked a variety of several professions. There is a common acceptance from the parties involved that might help the first steps be taken toward overcoming barriers, e.g., by developing a common framework for quality-assured intersectional cooperation in the field of CMD prevention in employees.

## 1. Introduction

Mental illness is responsible for the rising number of sickness-related absence and limited productivity at work, resulting in a significant loss of potential labor supply [1]. Around 20% of the working-age population in an average county of the Organisation for Economic Co-operation and Development (OECD) suffers from a clinical mental illness. Most of these illnesses are common mental disorders (CMDs). CMDs are the most frequent cause for long-term work disability [2] and early retirement [3]. Therefore, one of the main actual challenges for policy makers is to prevent mental health problems [1,4]. From a business perspective, investing in improving mental health at the workplace holds profit with a return on investment of up to 9€ for every 1€ spent [5]. Moreover, the workplace is a suitable place for prevention. Early interventions for CMDs offered at the workplace have been proven to be successful in addressing employees at risk [6], reducing sickness absence [7], and minimizing recurrent sickness absence [8]. These data emphasize the vital importance of collaboration between professions and sectors.

Unfortunately, this collaboration cannot be taken for granted. Research confirms the existence of major barriers in the cooperation of different relevant health professionals working in the field of prevention [9,10,11,12,13,14,15,16,17]. Preventive activities in the workplace setting often take place without any interactions with primary care physicians, psychotherapists, and rehabilitation facilities. International literature on the subject states that better cooperation among the key players involved in the health care system is the top political recommendation [18]. In some countries, guidelines in occupational health for the management of CMDs in employees do exist. However, many of those are poorly suited and rarely followed [19]. Although integrated structures and ways of cooperation such as interdisciplinary and cross-specialist health care supply networks have been developed in Germany, they are still very limited [20,21]. One example for an integrated health care service is the Psychosomatic Consultation in the Workplace (PSIW), which offers psychosomatic outpatient care at the workplace [6,22,23,24]. In this framework, psychotherapists are in close contact with the occupational health physician (OHP) as well as with important company professionals such as human resource managers (HRM). It has been shown that patients are more likely to contact PSIW at an earlier stage of disease [23] and are more satisfied [25] compared to the usual outpatient care. Unfortunately, primary care physicians (PCP) representing the primary care sector are usually not involved in this program on a regular basis.

In order to tackle these cooperation problems, it is necessary to become aware of the impediments in cooperation, to know the general attitudes among health care providers and vocational professionals toward the significance of CMDs and their prevention in employees, and to learn from the corresponding experiences. Systematic approaches to these topics have been missing so far. Therefore, we started the project with the acronym PHOEBE in 2014 [26] in order to gather information from the following four professional groups: occupational health physicians (OHPs), human resource managers (HRMs) in the vocational setting, primary care physicians (PCPs), and psychotherapists (PTs) in the outpatient care setting. We asked them about their perceptions and cooperation experiences in the prevention of CMDs in employees. In the following, we focus on the question of what these four groups think regarding which key players in companies, in the healthcare sector, and in the social security system should be involved in primary, secondary, and tertiary prevention (see Box 1 for definitions of the prevention fields).

Box 1 **Definition of prevention fields in the German health care system (adapted from [27]).**

***Primary prevention*** aims to prevent diseases before they occur. This includes preventing exposure to relevant hazards, altering unhealthy behavior, and increasing resistance to disease in case of exposure.

***Secondary prevention*** aims to reduce the impact of diseases that have already occurred. This includes detecting and treating a disease as soon as possible to halt or slow its progress.

***Tertiary prevention*** aims to soften the impact of an ongoing illness that has lasting effects. This includes helping people manage long-term and complex health problems to improve their quality of life as much as possible, as well as their ability to function and work.

## 2. Materials and Methods

### 2.1. Study Design and Sample

The complete study design and a detailed description of the study sample can be found in Michaelis et al. 2016 [26]. In short, in July 2014, a questionnaire was sent by mail to 1000 randomly selected PCPs and 700 randomly selected PTs from the Internet address database of statutory health care physicians and psychotherapists (www.arztsuche-bw.de). Further, it was sent to all OHPs (*N* = 450, member of a professional association) and HRMs (*N* = 1426, member of an employers’ association in the metal and electric industry) in the database of an employers’ association in the metal and electric industry in the South German federal state, Baden-Württemberg. Response rates were 30% (*N* = 133) for OHPs, 14% (*N* = 136) for PCPs, 27% (*N* = 186) for PTs, and 12% (*N* = 172) for HRMs.

The standardized questionnaire was self-administered and partly based on the results of our own qualitative research, e.g., at the interface of cooperation between general practitioners and occupational health physicians [9,15]. It included questions about attitudes and experiences in preventing CMDs in employees as well as professional and personal characteristics, our own knowledge/own activities in preventing CMDs in employees, and further experience in CMDs. 

The study was approved by the ethics committee of the medical faculty of the Eberhard Karls University Tübingen (204/2014/BO2).

The research question that we focus on in this contribution was operationalized as: in your opinion, who should play a key role in the prevention of common mental disorders (CMD) in employees? This question was followed by a short description of primary, secondary, and tertiary prevention, as shown in Box 1. The study participants were asked to check “yes”, “no”, or “I don’t know” for the eight defined potential key players for each prevention field. These key players were categorized according to the following three sectors:

a) vocational setting: OHPs, HRMs/superiors, employee representatives
b) outpatient care: PCPs, PTs
c) statutory insurance agents: statutory health, pension, and accident insurance

We also offered the possibility of adding further important key players, but no others were named. As the settings and responsibilities vary in different health care systems, we present a short description of every addressed key player in the vocational setting, in the outpatient care, and in the statutory insurance system below.

a) Professionals in the vocational setting
  *Occupational health physicians* are employed in every German workplace, as obliged by German law. OHPs offer workplace-related and sometimes general health services for employees. They deal with all work-related prevention issues [28], and therefore have interferences, e.g., with PCPs [9,13,14,15] in secondary prevention or with rehabilitation professionals [17] in tertiary prevention. They serve as mediators inside and outside the company [29]. Besides work-related health and safety issues, they are often at a central position of workplace health promotion. The (early) detection of CMDs and psychosomatic symptoms in employees may be covered by routine methods of OHPs’ work. In addition, OHPs have the possibility of obtaining further qualification, e.g., in psychosomatic primary care (’Psychosomatische Grundversorgung’ [30]) or in psychotherapy [31].
  *Human resource managers* manage the employees of a company. This is not reduced to hiring and firing, but also includes motivating and promoting employees as well as long-term commitment to the company and workplace health and safety issues. In detail, HRMs coordinate the range of company medical services with occupational health physicians and enable employees to make use of them. In smaller enterprises, the HRM is (one of) the superior(s). The important role and influence of superiors’ leadership patterns on employee job satisfaction, job well-being, sickness absence, and disability pensions is well documented [32,33].
  *Employee representatives* are elected by the employees to represent their interests. According to the German Act on Occupational Safety and Health (‘Arbeitsschutzgesetz’), all actions connected with occupational health and safety must be planned by the employer in cooperation with the employee representatives and OHPs. In addition, other relevant professionals for occupational health and safety are obliged to cooperate with them (German Occupational Safety Act, ‘Arbeitssicherheitsgesetz’).
b) Professionals in outpatient care
  *Primary care physicians*: In German health care, patients are encouraged to first consult their PCP with any health problem before seeking specialty treatment. Employees in Germany need to present a sick note from a physician to benefit from paid sick leave. The tasks of PCPs cover all fields of prevention, from primary prevention (e.g., vaccinations), early detection, the management and treatment of diseases, individual health-promoting, and lifestyle counseling to rehabilitation [34]. Psychosomatic primary care is an integral part of the continuous medical education of PCPs. Often, primary care physicians are the first professional contact for people with CMDs in Germany [35]. The majority of these patients are treated solely by them, e.g., via regular consultations that include counseling, problem-solving, and sometimes cognitive–behavioral techniques [36].
  *Psychotherapists*: Psychotherapy is delivered by physicians who specialize in psychiatry or psychosomatic medicine, or by psychologists who are especially trained in psychotherapy. They work in inpatient and outpatient care. The majority of psychotherapy consultations is offered in outpatient care [35,37]. In this contribution, we refer to PTs working as practitioners in outpatient care.
c) Statutory insurance agents
  *Statutory health insurance agents*: Covering more than 90% of the population, they are the central pillar of the German health care system. They have a comprehensive legal mandate for health promotion and prevention, and offer primary prevention in the vocational setting, partly in cooperation with the statutory accident insurance [38,39].
  *Statutory pension insurance agents*: They provide medical rehabilitation with the goal of regaining participation in working life as well as preventive strategies with a special emphasis on work-related health risks. They develop innovative programs with partners in companies and the public health sector [40]. One of these German programs in cooperation with OHPs is called BETSI (‘Ensure employability participatory oriented’), offering early preventive strategies for employees [41].
  *Statutory accident insurance agents* contribute to the prevention of accidents at work, occupational diseases, and work-related health hazards. If necessary, it also contributes to the recovery of the health and performance of the insured with all eligible funds and makes compensation payments to them or their dependents. In a recent position paper, the Federation of the German Statutory Accident Insurance addressed the prevention of work-related mental and psychosocial stress. Here, the current priorities are seen particularly in: supporting systematic risk assessments, strengthening the resources of companies and employees, qualifying managers, providing practical guidelines, and strengthening the cooperation with statutory health and pension insurance agents [42]. This is implemented in the work program Protection and Strengthening of Health at Work-Related Mental Load [43].

### 2.2. Statistical Methods

Descriptive measures calculating the mean, standard deviation, and relative frequencies were included in the analysis if appropriate. 

One-quarter (25%) of respondents missed marking at least one item. A non-response of all eight items within a prevention field was seldom (1%, 3%, and 6%, respectively in primary, secondary, and tertiary prevention). One item within a prevention field was missing in 6%, 2%, and 3% respectively. The answer “I don´t know” was more frequently given when assessing statutory insurance agents (primary prevention: statutory health insurance agents [PTs, OHPs 14%], secondary prevention: statutory pension and accident insurance agents [all groups, 11–19%], and tertiary prevention: statutory accident insurance agents [PCPs, PTs, HRMs, 11–19%]). “I don´t know” and missing answers were generally interpreted as “no, no particular engagement required”.

Based on the descriptive statistics, the non-parametric McNemar test with IBM SPSS 22 (SPSS Inc., Chicago, IL, USA) was used to decide if the most frequently marked relevant professional was addressed as often as the following one. This was done by focusing on non-significant differences (*p* > 0.05) and separately for each professional group and each prevention field. 

## 3. Results

Male respondents included 53% of OHPs, 50% of PCPs, 30% of PTs, and 58% of HRMs. Of the latter group, 18% were executive directors or owners. Further, 54% of HRMs worked in large enterprises with more than 250 employees, 38% worked in medium-sized enterprises (<250 employees), and 8% worked in small enterprises (<50 employees).

Nearly all OHPs, three out of four PCPs, and every second PT and HRM had experience with return-to-work programs for employees with long-term sick leave. One out of four OHPs and one out of three PCPs reported experience with psychiatric/psychosomatic care during their medical specialization. The majority of the respondents had long occupational experience (see Table 1).

Amongst the professional groups considered in this study, OHPs were regarded to have the biggest potential to influence any prevention field of CMDs in employees. The following results can be summarized with regard to the different prevention fields (for detailed information, see Table 2):

- In primary prevention, HRMs regarded themselves, OHPs, and health insurance agents as equally relevant as key players for prevention, in relation to percentages. PTs indicated an important role for employee representatives in this field.
- In secondary prevention, PCPs were regarded to be as relevant as OHPs. HRMs again indicated themselves as equally responsible as OHPs and PCPs.
- In tertiary prevention, only OHPs identified themselves as main protagonists. The other groups marked several professions: PTs and PCPs committed OHPs, PTs, PCPs, and pension insurance agents. PTs also marked health insurance agents. HRMs indicated a relevant role for PTs and for health insurance agents next to OHPs.

Table 2 shows the exact percentages attributed to defined key players in the prevention of CMDs in accordance with the McNemar test. 

## 4. Discussion

To the best of our knowledge, this is the first multi-professional study investigating the views of 627 health professionals in the prevention of CMDs in employees, namely occupational health physicians (OHPs), primary care physicians (PCPs), psychotherapists (PTs), and human resource managers (HRMs). Knowing their opinions is the premise for improving coordination and communication. However, this is one of the biggest challenges the health care system is facing today in the prevention of CMDs [1,18]. 

The first result indicates a substantial agreement regarding the importance of OHPs in the commitment to prevention. This corresponds to the self-description of professional associations of OHPs [29,44]. OHPs can contribute to primary prevention if HRMs and superiors follow their recommendations. They are capable of detecting CMDs in employees at an early stage (secondary prevention), but they need to refer the patients to PCPs and PTs for further treatment. According to our survey, they are, moreover, the professional group most often involved in return to work. However, in order to successfully return to work, communication between PCPs, PTs, and HRMs/superiors is often required. Cooperation seems to be the key, as the evaluation of recently established projects show promising results. For example, the concept Psychosomatic Consultation in the Workplace (PSIW) [6,22,24] connects PTs, OHPs, and HRMs, and initial results show that more patients at risk are reached [23]. In addition, patients using PSIW are more satisfied with the consultation [25] compared to the usual outpatient care. 

Primary care physicians (PCP) are often not included in interventions of work-related mental health care. In previous research, we found out that OHPs often face resistance when trying to interact with other medical groups [9,10,11,12,13,14,15,16,17,45,46]. Another part of our survey covered collaboration [47]. We found that 70% to 90% of OHPs reported first contact with a PCP or PT, while only 40% of PCPs and 33% of PTs approached an OHP. This underlines the need for new ways of cooperation between relevant key players both in the vocational setting and the health care system.

The key role of OHPs indicates the need for an enlarged standard training in the field of prevention and treatment of CMDs. Indeed, there are ways to deepen knowledge through special training. A special qualification of OHPs is positively correlated with the existence of vocational prevention programs for CMDs [48]. Still, this training depends on the interest and motivation of every single OHP, and is not yet established as an important standard criterion. 

The second result is the high commitment of HRMs to all fields of prevention. Research highlights the connection between leadership style and employee health [32,49]. HRMs and superiors have more influence on workplace factors than any other professional in this field. Public awareness has increased within the last years, e.g., in the form of information strategies [50,51,52], practical guidelines, and the working tools of diverse national and international initiatives [43,53,54,55]. Having this in mind, it is astonishing that medical professionals rated the role of HRMs/superiors rather low. Our research results and others in the field of leadership underline the importance of promoting good leadership in general, as well as developing standardized, qualitative trainings that address the handling of CMDs for HRMs and superiors.

One more interesting result in the field of primary prevention is that PTs assign employee representatives a relevant role for the commitment to primary prevention. Employee representatives participate in decision making especially when it comes to issues that affect employee health and safety. Although they are also not especially trained in the field of CMDs, they are often approached by employees when it comes to trouble with their superior, which in turn makes them a relevant key player for prevention. Cooperation between employee representatives and superiors is a delicate topic as well, but would exceed the scope of this paper [56]. 

In secondary prevention, PCPs are perceived to be as relevant as OHPs. This corresponds to their role in the German health care system as the first contact person and gatekeeper, referring patients to secondary care if necessary [34]. Thus, patients suffering from CMD are part of their daily routine. On the other hand, studies show low recognition rates, i.e., of maximum 50% in generalized anxiety disorder [57]. Rates were even lower if patients presented physical symptoms. Similar to OHPs, PCPs have opportunities for special training. However, in contrast to OHPs, qualification entitles PCPs to bill short psychosocial interventions, which are called psychosomatic primary care. Although this approach was shown to be effective [36], PCPs have many tasks and only limited time [58]. Again, there seems to be an urgent need for PCPs to deepen their knowledge of CMDs (it is not enough that only personally motivated PCPs invest time and money in special trainings) as well in order to improve cooperation with other relevant health professionals.

In tertiary prevention, PTs and insurance agents are ranked at the top. This goes in line with other findings [59,60,61], where medical professionals rate the work of PTs in CMDs as helpful. To our knowledge, HRMs/superiors have not been asked yet.

As described under Materials and Methods, the main task of pension insurance agents is to support patients suffering from CMD in participating in working life again. This corresponds to the definition of tertiary prevention. Health insurance agents offer mainly primary and secondary prevention. It is unclear why they were considered to be important in tertiary prevention. HRMs might remember them first because they regularly work together in primary prevention [39], whereas they normally have little contact with pension insurance agents. Interestingly, accident insurance agents also play an official role in prevention [38,39], but are remembered least. In our opinion, this states a general need for more information and sensitization of the role of statutory insurance agents.

Compared to primary and secondary prevention, the ranking in tertiary prevention seems to be not that meaningful, since several key players are being viewed as equally relevant. This indicates the need to establish ways of cooperation between all relevant health professions in this field, including insurance agents, when it comes to bringing employees with CMDs back to work. Our recent study might give some indications on how to improve cooperation between different stakeholders in the field of health and employment. In addition, it points out that there is a lack of information on the significance, competencies, and responsibilities of the individual professionals. Some programs exist [40,41] that prove the efficiency of network-based acting strategies for OHPs, PCPs, and specialist clinics as well as interdisciplinary cooperation [21,62]. Systematic evaluation and a broad roll-out are missing. 

## 5. Limitations

One limitation is the very low questionnaire response rate in the sample of PCPs and HRMs (12% and 14%, respectively). The low response rates in HRMs correspond to our other investigations, especially in small enterprises [13,63,64]. The response rate of 27% among PTs is low compared to recent other German studies with rates up to 50% [65,66], while the response rate of OHPs meets the expectations [63,67]. 

Although response rates were low, absolute numbers of participants are still high in the field. Furthermore, it is strength of the study to approach a genuinely qualitative question with a systematic, quantitative approach. Looking at the sample characteristics [26], important fields seem to be covered. Participating PCPs were working in equal parts in the city, in the city periphery, and in the countryside. Participating OHPs were working mainly (57%) in the city, which corresponds to a higher density of employees in cities than in the countryside. Only 30% of PTs were male, which corresponds to the female predominance in this field. HRMs were mostly from large (<249 employees, 54%) and medium-sized companies (50–250 employees, 38%), while small companies (<50 employees, 8%) were underrepresented, and women were overrepresented (42% female). This might lead to a positivity bias, as studies about barriers for workplace health promotion have shown that many superiors in small enterprises still seem to avoid the question of responsibility for prevention [68,69]. Finally, the exploratory and descriptive methods used in this study allow no attributions of causality.

## 6. Conclusions

With respect to the increasing burden of diseases in the field of CMDs, such as productivity losses and rising costs, prevention must be intensified in the future. This is especially true for primary prevention issues in the workplace setting with the need for a holistic health management especially in small and medium-sized companies. In the secondary and tertiary field, an interdisciplinary cross-linked care is considered optimum [62], but this is still out of reach. Still, the common view of relevant professionals as shown in our survey might be seen as a basis for overcoming barriers in the intersectional cooperation between vocational setting and the health care system [70]. A consensus framework for quality-assured intersectional cooperation in the prevention of CMDs in employees is urgently needed.

## Figures and Tables

**Table 1:** Sample characteristics.

Variables	Title	Mean	SD	Min–Max	*N*
Age	Occupational health physicians	54.9	8.0	36–77	133
	Primary care physicians	53.7	8.6	37–75	130
	Psychotherapists	53.9	8.6	31–71	183
	Human resource managers	48.8	8.1	25–66	159
Years in profession as OHP or PCP	Occupational health physicians	26.7	8.5	8–50	114
	Primary care physicians	18.3	9.3	2–35	127
Years of experience in outpatient practice as PT	Psychological psychotherapists	13.1	8.8	0–34	116
	Physicians working as psychotherapist	14.3	7.2	1–31	66
Years of named professional position as HRM	Executive directors/owners of the enterprise	15.2	8.7	1–31	28
	Human resource managers	9.7	8.2	0–38	120

Legend: *N* = 627 (133 OHPs, 136 PCPs, 186 PTs, 172 HRMs). Abbreviations: HRM = human resource manager; PCP = primary care physician; OHP = occupational health physician; PT = psychotherapist (outpatient care); SD = standard deviation.

**Table 2:** Who should play a key role in the prevention of common mental disorders in employees? Perceptions of four professional groups.

Prevention Field	Key Players in Prevention of CMDs of Employees	% of Approval by Professional Group			
		OHPs	HRMs	PTs	PCPs
Primary prevention	Occupational health physicians	**89.5**	**68.6**	**83.3**	**91.2**
	Psychotherapists	15.8	21.5	38.7	16.9
	General practitioners	59.4	53.5	50.0	64.0
	Employee representatives	74.4	48.8	**80.6**	75.7
	HRMs/superiors	81.2	**70.9**	74.7	77.2
	Health insurance agents	56.4	**66.9**	53.8	44.1
	Pension insurance agents	34.6	24.4	33.9	36.0
	Accident insurance agents	55.6	39.0	42.5	44.1
Secondary prevention	Occupational health physicians	**88.7**	**76.7**	**86.0**	**84.6**
	Psychotherapists	68.4	49.4	75.3	66.2
	General practitioners	84.2	**69.8**	**82.3**	**84.6**
	Employee representatives	55.6	43.6	62.9	69.1
	HRMs/superiors	71.4	**69.2**	61.8	64.0
	Health insurance agents	62.4	61.6	55.9	54.4
	Pension insurance agents	54.9	27.9	41.9	49.3
	Accident insurance agents	48.1	37.8	43.5	42.6
Tertiary prevention	Occupational health physicians	**87.2**	**70.3**	**77.4**	**82.4**
	Psychotherapists	79.7	**76.2**	**80.1**	**79.4**
	General practitioners	76.7	65.1	**76.9**	**82.4**
	Employee representatives	63.9	51.2	68.3	67.6
	HRMs/superiors	72.9	68.6	66.1	71.3
	Health insurance agents	75.2	**73.8**	**75.3**	72.1
	Pension insurance agents	82.0	64.0	**79.0**	**75.0**
	Accident insurance agents	65.4	52.9	62.9	56.6

Legend: *N* = 627 (133 OHPs, 136 PCPs, 186 PTs, 172 HRMs). The columns indicate how many of the four surveyed professional groups marked the defined key player as relevant for prevention. Key players with the highest approval (‘yes’ vs. ‘I don´t know’/missing) are marked red and set in bold. If several key players are marked within one column, no statistically significant difference was found applying the McNemar test. If only one value is marked within a column, the McNemar test revealed a statistically significant difference in comparison with the other values. Abbreviations: CMDs = common mental disorders; OHPs = occupational health physicians; HRMs = human resource managers; PTs = psychotherapists; PCPs = primary care physicians.
